# Association between magnesium, erythropoietin resistance and mortality: the Japanese Dialysis Outcomes and Practice Patterns Study (J-DOPPS)

**DOI:** 10.1093/ckj/sfae153

**Published:** 2024-06-21

**Authors:** Sawako Kato, Jui Wang, Yoshihiro Onishi, Masaomi Nangaku

**Affiliations:** Department of Nephrology, Nagoya University Graduate School of Medicine, Nagoya, Japan; Institute for Health Outcomes and Process Evaluation Research (iHope International), Kyoto, Japan; College of Public Health, National Taiwan University, Taipei, Taiwan; Institute for Health Outcomes and Process Evaluation Research (iHope International), Kyoto, Japan; Division of Nephrology and Endocrinology, The University of Tokyo Graduate School of Medicine, Tokyo, Japan

**Keywords:** erythropoietin resistance index, hemodialysis, magnesium, mortality, renal anemia

## Abstract

**Background:**

Limited data are now available to evaluate the relationship between serum magnesium level, anemia and mortality in the dialysis population.

**Methods:**

Using data from the Japanese Dialysis Outcomes and Practice Patterns Study (J-DOPPS) phases 5 and 6, we analyzed the association between serum magnesium (s-Mg) levels and the erythropoiesis-stimulating agents resistance index (ERI) as the primary outcome. To estimate the longitudinal relationship, a mixed-effect model was used with ERI at each 4-month period as the dependent variable and quintiles of s-Mg at the previous 4-month period as the independent variable. We also examined incidence of infectious events, and the all-cause and cardiovascular disease (CVD)-related deaths as secondary outcomes by Cox regression with quintiles of s-Mg at baseline.

**Results:**

Of the 4776 participants in J-DOPPS, 1650 were included in the analysis. The median of s-Mg at baseline was 2.5 mg/dL. A significant linear association of s-Mg with ERI (*P* for trend <.001) was revealed. Low and high s-Mg levels were not associated with the clinical outcomes of interest, except for the highest quintile of s-Mg being significantly associated with lower incidence of all-cause mortality and CVD-related deaths compared with the middle (reference) quintile.

**Conclusions:**

We observed that lower s-Mg levels subsequently induced higher ERI and that mild higher s-Mg levels were possibly associated with good rather than poor outcomes in Japanese hemodialysis patients. Adjustment of s-Mg levels may be proposed as a new strategy at a low cost and risk to reduce the risk of premature mortality.

## Graphical Abstract

**Graphical Abstract:**
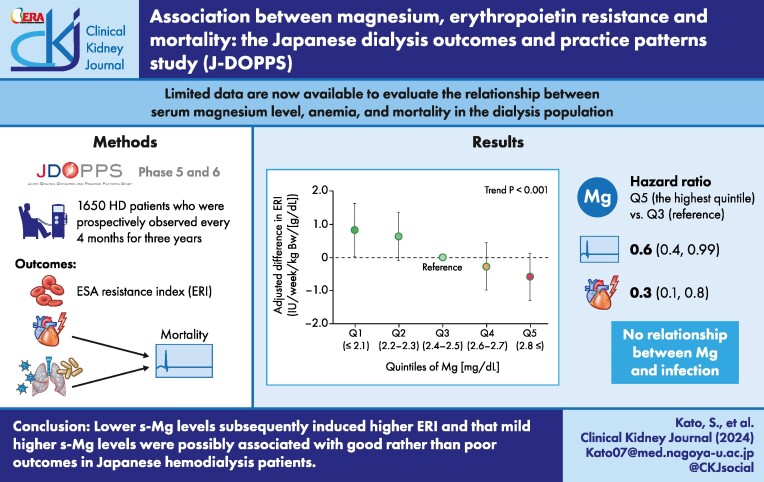

KEY LEARNING POINTS **What was known:**

- Excessive hypermagnesemia is still life-threatening, although there have been increasing reports of an association between poor outcome and hypomagnesemia in diverse clinical situations.
- We aimed to clarify the impact of hypomagnesemia on renal anemia, infections and cardiovascular mortality in hemodialysis patients.

**This study adds:**

- We examined 1650 hemodialysis patients enrolled in this large-scale cohort study.
- Lower serum magnesium (s-Mg) levels subsequently induced higher erythropoiesis-stimulating agents resistance index.
- Mild higher s-Mg levels were possibly associated with good rather than poor mortality.

**Potential impact:**

- Our results highlighted the importance of delicate control of magnesium level in dialysis management.

## INTRODUCTION

The global dialysis population continues to rise and perturbing questions remain unsolved, especially the high burdens of comorbidities and symptoms and concomitant low health-related quality of life [1]. Despite technical improvements in hemodialysis, patients undergoing dialysis still have a higher risk for premature death than the general population [2]. The high premature mortality in these patients is due not only to cardiovascular disease (CVD) but also to non-CVD causes of death, especially infectious complications [3]. CVD and infectious diseases seem to potentiate each other [4], possibly because they share risk factors and potential causal pathways [5, 6]. Renal anemia is a common complication of chronic kidney disease (CKD), and a low hemoglobin concentration is still the leading risk factor for high mortality, even with the widespread use of erythropoiesis-stimulating agents (ESAs) [7].

During the 1990s, the approval and increasing prevalence of ESA use undeniably caused a revolutionary improvement in the management of renal anemia in CKD patients [8]. However, there is a variability in sensitivity to these drugs in CKD patients and up to 10% of patients receiving ESA show poor responsiveness to ESA [9]. Three well-known randomized controlled trials to investigate optimal hemoglobin targets for the reduction of CVD mortality revealed that patients in the high-hemoglobin group failed to show superiority; indeed, increased mortality was found in the ESA high-dose group [10–12]. Besides, several factors—iron deficiency, inflammation, poor nutrition, uncontrolled hyperparathyroidism—are recognized as causing ESA resistance [13], which suggests that ESA resistance may also share risk factors for higher premature mortality.

Many studies have reported that hypomagnesemia is associated with increased mortality in dialysis patients [14, 15]. Magnesium (Mg) plays a key role in mineral and bone disorders in CKD (CKD-MBD) in conjunction with phosphate and calcium, and its deficiency is associated with accelerated vascular calcification and increased CVD [16]. Mg deficiency is also known to be linked to immune dysfunction, inducing lasting low-grade inflammation, as well as to susceptibility to infectious diseases [17]. It has been reported recently that hypomagnesemia is associated with anemia in pre-dialysis CKD patients and with ESA resistance in hemodialysis patients [18]. However, data to evaluate the relationship between serum Mg level, anemia and mortality in the dialysis population remain limited. In this study, we sought to clarify the impact of hypomagnesemia on ESA resistance, infections, CVD-related mortality and all-cause mortality in patients enrolled in the Japanese Dialysis Outcomes and Practice Patterns Study (J-DOPPS).

## METHODS

### Data sources

We used data from J-DOPPS, a part of the worldwide DOPPS, in which adult hemodialysis patients were randomly enrolled from representative dialysis facilities in each country and were prospectively observed every 4 months for 3 years. Details of DOPPS design have been published elsewhere [19]. J-DOPPS was approved by the Ethics Committee of Tokyo Women's Medical University (approval number 2388 and 2388-R4).

### Study participants

In the present study, we used data from J-DOPPS phases 5 (2012–15) and 6 (2015–18). We included patients who received an ESA at the start of observation (baseline). We excluded patients if they had had a history of malignancy, gastrointestinal bleeding, liver cirrhosis, sickle-cell anemia or transfusion, and if a serum Mg (s-Mg) value at baseline was not available.

### Exposure and outcomes

The exposure of interest was s-Mg. To delineate the relationship between s-Mg and outcomes, we used quintiles (Q1–Q5) of the baseline distribution of s-Mg.

The outcome of primary interest was the ESAs resistance index (ERI), defined as the weekly erythropoietin dose (IU/week) per body weight (kg) per blood hemoglobin concentration (g/dL). We calculated ERI from the average erythropoietin dose and the average hemoglobin concentration in each 4-month period of observation. Weekly doses of long-acting ESAs were converted to erythropoietin dose equivalent by multiplying by 200 for darbepoetin alpha [20] and by 225 for epoetin beta pegol [21].

We also examined the incidence of infection and CVDs as secondary outcomes including: (i) deaths due to infectious disease, (ii) hospitalizations due to infectious disease, (iii) diagnosis of bacteremia, (iv) all-cause mortality, (v) mortality due to CVD and (vi) sudden death. The infectious diseases included pneumonia, sepsis, endocarditis, AIDS, urinary tract infection, trauma infection, abscess, cellulitis, osteomyelitis, viral infection and fungal infection. The CVDs included angina, acute myocardial infarction, cardiac arrest, heart failure, ischemic stroke, ischemic brain injury, transient ischemic attack, pulmonary thromboembolism, mesenteric infarction and deep vein thrombosis. The date of incidence of these outcomes was identified for each patient, and the survival period was defined from the baseline date to either the outcome of interest or the end of follow-up, whichever came earlier.

### Statistical analysis

To describe patient characteristics, continuous variables were summarized as mean and standard deviation or median and interquartile range (IQR), and categorical variables as proportions.

To estimate the longitudinal relationship between s-Mg and ERI, a mixed-effect model was used with ERI at each 4-month period as the dependent variable, quintiles of s-Mg at the previous 4-month period as the independent variable, patients as random effects, the 4-month period as a repeated index, and age, sex, dialysis vintage, diabetic nephropathy, body mass index (BMI), serum albumin, conventional or long-acting ESA type, and the use of iron drugs (oral iron, intravenous iron and iron-containing phosphate binders) as covariables. The results were summarized as a *P*-value for the trend. To present the shape of the relationship, point estimates and 95% confidence intervals (CI) in difference of ERI by s-Mg quintiles were determined. To explore whether the effect of s-Mg on ERI is mediated by bone disease and/or inflammation, we did an additional analysis using a regression model with intact parathyroid hormone and C-reactive protein added to the covariables of the model mentioned above.

To delineate the relationship between different s-Mg levels and clinical events, crude and adjusted Cox regression analyses with quintiles of s-Mg at baseline as an independent variable were used to estimate the hazard ratio (HR) and 95% CI. In the adjusted analyses, we selected the following potential confounders: age, sex, BMI, cause of end-stage kidney disease, dialysis vintage, type of vascular access, comorbid coronary artery disease and diabetes mellitus, single-pool Kt/V, serum levels of creatinine, albumin, phosphorus, intact parathyroid hormone, C-reactive protein, ferritin, calcium, leukocytes, conventional or long-acting ESA type, ERI and the use of iron drugs.

We accounted for missing data by using the multivariate imputation by chained equation (MICE) algorithm with the predictive mean matching method for all types of variables and Rubin's rule. Statistical analyses were performed using SAS version 9.4 (SAS Institute, Cary, NC, USA).

## RESULTS

### Patient characteristics

**Figure 1:**
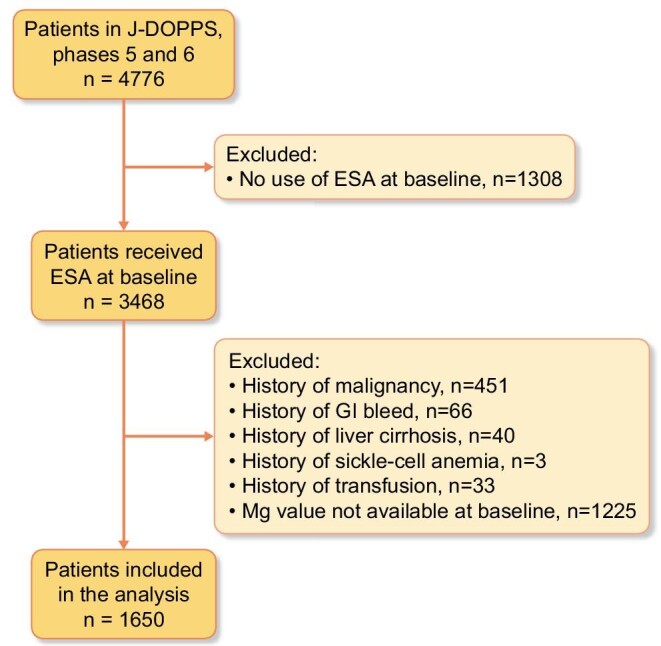
Flow diagram of patient inclusion. Mg, serum magnesium concentration.

Of the 4776 J-DOPPS participants, 1650 were included in the analysis (Fig. 1). Table 1 shows the characteristics of the subjects analyzed. The mean age was 66 years; 39% were female. They had a median hemoglobin of 10.7 g/dL (IQR 10.0, 11.4) and a median ESA dose of 4339 IU/week (IQR 2500, 7225). Compared with patients excluded from analysis due to a missing s-Mg value, the analyzed population had a slightly longer history of dialysis, more comorbid CVD, more arteriovenous fistulas in vascular access, less use of iron preparations, lower serum ferritin and more prescriptions of proton pump inhibitors (PPIs).

**Table 1:** Baseline characteristics of the study patients.

	Study patients (*N* = 1650)		Patients excluded due to missing s-Mg value (*N* = 1225)	
Items	Summary statistics	Number of missing values	Summary statistics	Number of missing values
Age (years)	65.6 (12.1)	0	65.2 (12.3)	3
Female sex (%)	38.8	1	33.7	1
Primary cause of end-stage kidney disease (%)		0		0
Diabetic nephropathy	38.8		38.9	
Chronic glomerulonephritis	32.2		32.4	
Others	29.0		28.8	
Hemodialysis vintage (years)	4.2 (1.0, 10.3)	2	3.8 (1.1, 8.8)	6
BMI (kg/m^2^)	21.7 (3.8)	68	21.7 (3.6)	128
Comorbidities (%)		0		0
Diabetes	42.6		41.1	
Cardiovascular diseases	57.2		50.1	
Vascular access (%)		0		0
Arteriovenous fistula	88.9		83.8	
Others	11.1		16.2	
Single pool Kt/V	1.4 (0.3)	137	1.4 (0.3)	237
Dialysate Mg concentration (mEq/L)	1.0 (1.0, 1.0)	71	1.0 (1.0, 1.0)	196
Laboratory				
Serum albumin (g/dL)	3.6 (0.4)	5	3.7 (0.4)	80
C-reactive protein (mg/dL)	0.1 (0.1, 0.4)	391	0.1 (0.1, 0.4)	517
Serum calcium (mg/dL)	8.8 (0.7)	80	8.8 (0.8)	116
Serum potassium (mEq/L)	4.8 (0.7)	9	4.8 (0.7)	13
Leukocytes (10^3^/mL)	5.8 (2.0)	212	6.0 (1.9)	74
Anemia-related items				
Hemoglobin (g/dL)	10.7 (1.1)	5	10.6 (1.2)	11
Serum ferritin (ng/mL)	65.4 (31.5, 139.0)	188	83.3 (38.0, 180.9)	330
ESA dose (units/week)	4339 (2500, 7225)	0	4426 (2586, 6942)	0
Use of iron drugs (%)	31.9	0	35.2	0
Medications (%)				
PPIs	47.2	0	42.0	0
Magnesium oxide laxatives	1.8	0	2.7	0

Data are presented as mean (standard deviation), median (IQR) or percentage.

### Serum magnesium concentration

**Figure 2:**
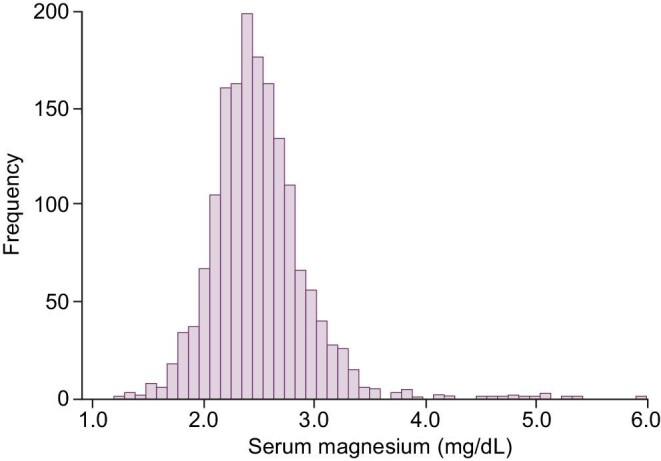
Distribution of serum magnesium at baseline in the study subjects.

The distribution of s-Mg at baseline was symmetrical with a median of 2.5 mg/dL (IQR 2.2, 2.8), as shown in Fig. 2. The proportion of subjects having s-Mg below the lower reference limit of 1.8 was 2.3%.

### Outcomes

Baseline median ERI was 7.4 IU/week/kg Bw/[g/dL] (IQR 4.3, 12.4).

During a total of 3353 person-years of observation, the incidence of each clinical event (/100 person-years) was as follows: death due to infectious disease, 1.0; hospitalization due to infectious disease, 4.8; diagnosis of bacteremia, 1.5; all-cause mortality, 4.6; deaths due to CVD, 2.2; and sudden death, 0.8.

### Relationship to ERI

**Figure 3:**
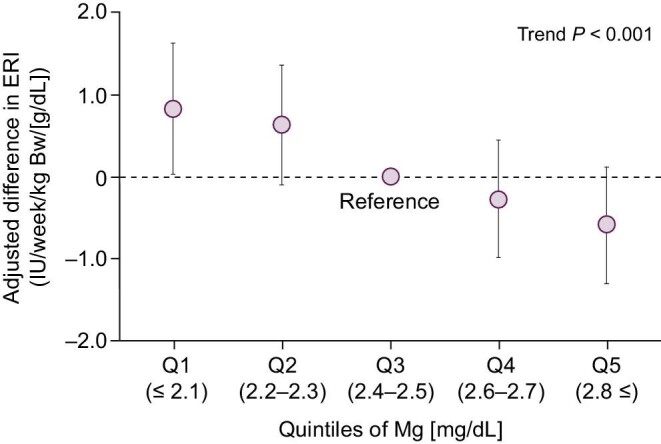
Model-adjusted relationship between ERI and quintiles of serum magnesium (Mg). Point estimates and 95% CI of between-group difference with Q3 as the reference are presented.

The mixed model analysis revealed a significant association between s-Mg and ERI (*P* for trend <.001). Figure 3 delineates the linear relationship between s-Mg and ERI.

The additional model analysis with intact parathyroid hormone and C-reactive protein added to covariables showed a very similar relationship between s-Mg and ERI (Supplementary data, Fig. S1).

### Relationships of clinical events

The relationships between s-Mg quintiles and the incidence of infectious events are shown in Table 2. For no outcome was there any apparent association between lower s-Mg and the incidence of infectious events.

**Table 2:** Crude and adjusted relationship between the incidence of infectious events and s-Mg.

	Mg quintiles	Description	Crude analysis	Adjusted analysis^a^
Outcome	(mg/dL)	IR (95% CI)	HR (95% CI)	HR (95% CI)
Deaths due to infectious disease	Q1 (≤2.1)	1.5 (0.8, 3.0)	1.1 (0.4, 2.7)	0.3 (0.1, 1.2)
	Q2 (2.2–2.3)	1.1 (0.5, 2.2)	0.8 (0.3, 2.0)	0.8 (0.3, 2.2)
	Q3 (2.4–2.5)	1.4 (0.8, 2.5)	Reference	Reference
	Q4 (2.6–2.7)	0.1 (0.0, 1.0)	0.1 (0.0, 0.8)	0.1 (0.0, 0.8)
	Q5 (2.8≤)	0.8 (0.4, 1.7)	0.6 (0.2, 1.5)	0.5 (0.2, 1.3)
Hospitalizations due to infectious disease	Q1 (≤2.1)	6.6 (4.7, 9.3)	1.4 (0.9, 2.2)	1.2 (0.7, 2.0)
	Q2 (2.2–2.3)	4.6 (3.2, 6.6)	1.0 (0.6, 1.6)	0.9 (0.6, 1.5)
	Q3 (2.4–2.5)	4.7 (3.4, 6.6)	Reference	Reference
	Q4 (2.6–2.7)	3.7 (2.5, 5.5)	0.8 (0.5, 1.3)	0.8 (0.4, 1.3)
	Q5 (2.8≤)	4.5 (3.3, 6.2)	1.0 (0.6, 1.5)	0.9 (0.6, 1.4)
Diagnosis of bacteriemia	Q1 (≤2.1)	2.5 (1.4, 4.3)	1.9 (0.8, 4.4)	1.4 (0.5, 3.4)
	Q2 (2.2–2.3)	1.4 (0.7, 2.6)	1.1 (0.4, 2.6)	1.2 (0.5, 3.0)
	Q3 (2.4–2.5)	1.3 (0.7, 2.4)	Reference	Reference
	Q4 (2.6–2.7)	0.9 (0.4, 2.0)	0.7 (0.3, 1.9)	0.6 (0.2, 1.8)
	Q5 (2.8≤)	1.5 (0.9, 2.6)	1.2 (0.5, 2.7)	1.1 (0.5, 2.7)

The 95% CI included 1.0 in all model-adjusted HR estimates.

a Adjusted for: age, sex, BMI, cause of end-stage kidney disease, dialysis vintage, type of vascular access, comorbid coronary artery disease and diabetes mellitus, single-pool Kt/V, serum concentrations of creatinine, albumin, phosphorus, intact parathyroid hormone, C-reactive protein, ferritin and calcium, and leukocytes, conventional or long-acting ESA type, ESAs resistance index and the use of iron drugs.

Mg, serum magnesium; IR, incidence rate (/100 person-years).

Table 3 shows the relationships between s-Mg and the incidence of CVD-related deaths. The highest quintile of s-Mg was significantly associated with a lower incidence of all-cause mortality and of CVD events compared with the middle (reference) quintile of s-Mg, although no apparent trend was noted.

**Table 3:** Crude and adjusted relationship between the incidence of CVD-related deaths and s-Mg.

		Description	Crude analysis	Adjusted analysis^a^
Outcome	Mg quintiles (mg/dL)	IR (95% CI)	HR (95% CI)	HR (95% CI)
All-cause deaths	Q1 (≤2.1)	5.1 (3.5, 7.4)	0.9 (0.6, 1.5)	0.6 (0.4, 1.1)
	Q2 (2.2–2.3)	6.3 (4.7, 8.5)	1.2 (0.8, 1.8)	1.2 (0.8, 1.8)
	Q3 (2.4–2.5)	5.4 (4.0, 7.3)	Reference	Reference
	Q4 (2.6–2.7)	4.1 (2.8, 5.9)	0.8 (0.5, 1.2)	0.8 (0.5, 1.3)
	Q5 (2.8≤)	2.8 (1.9, 4.2)	0.5 (0.3, 0.9)	0.6 (0.4, 0.99)^b^
Deaths due to CVDs	Q1 (≤2.1)	2.3 (1.3, 4.0)	0.8 (0.4, 1.7)	0.7 (0.3, 1.5)
	Q2 (2.2–2.3)	3.5 (2.3, 5.2)	1.3 (0.7, 2.3)	1.4 (0.7, 2.5)
	Q3 (2.4–2.5)	2.8 (1.8, 4.2)	Reference	Reference
	Q4 (2.6–2.7)	2.0 (1.2, 3.4)	0.7 (0.4, 1.5)	0.8 (0.4, 1.6)
	Q5 (2.8≤)	0.8 (0.4, 1.7)	0.3 (0.1, 0.7)	0.3 (0.1, 0.8)^b^
Sudden deaths	Q1 (≤2.1)	0.9 (0.4, 2.3)	1.5 (0.4, 5.3)	0.9 (0.2, 3.7)
	Q2 (2.2–2.3)	1.4 (0.7, 2.6)	2.2 (0.7, 6.5)	2.5 (0.8, 7.9)
	Q3 (2.4–2.5)	0.6 (0.3, 1.5)	Reference	Reference
	Q4 (2.6–2.7)	0.6 (0.2, 1.6)	0.9 (0.3, 3.5)	1.1 (0.3, 4.1)
	Q5 (2.8≤)	0.5 (0.2, 1.2)	0.7 (0.2, 2.7)	0.9 (0.2, 3.6)

a Adjusted for: age, sex, BMI, cause of end-stage kidney disease, dialysis vintage, type of vascular access, comorbid coronary artery disease and diabetes mellitus, single-pool Kt/V, serum concentrations of creatinine, albumin, phosphorus, intact parathyroid hormone, C-reactive protein, ferritin and calcium, and leukocytes, conventional or long-acting ESA type, ESAs resistance index and the use of iron drugs.

b With the sole exception of the association of the fifth quintile of Mg with all cause deaths and deaths due to CVD, the 95% CI included 1.0 in all model-adjusted HR estimates.

Mg, serum magnesium; IR, incidence rate (/100 person-years).

## DISCUSSION

In this study, we used data from the patients enrolled in the phase 5 and 6 of J-DOPPS, a large-scale long-term observational hemodialysis cohort in Japan. The present study is the first to simultaneously investigate the impact of Mg levels on three representative dialysis risk factors (anemia, CVD and infectious events) and mortality. We found that lower s-Mg levels were associated with worse ERI in subsequent months, but we found no apparent relationships between s-Mg levels and infectious events, CVD or mortality—with one exception: we found that the patients in the highest quintile of s-Mg levels showed significant decreases in all-cause mortality and in incidence of CVD death compared with the patients in the middle (reference) quintile.

Since Mg is the second-most abundant cation after potassium in the cells and essential for human health as a cofactor in numerous biological processes, disturbances in Mg levels could conceivably affect health in many different ways throughout the body [22]. Chronic Mg deficiency induces chronic inflammation, oxidative stress, insulin resistance and osteoporosis, and increases the incidence of diabetes mellitus [23], infectious disease [17] and CVD [24]. Mg plays a role in CKD-MBD [16]; vascular aging due to disturbance in Mg is suspected to occur prematurely and be more severe in CKD patients than in the general population. In a cohort study that compared patients with CKD with persons without CKD, an inverse association was found between s-Mg levels and mortality in both groups, while the CKD patients with hypomagnesemia (under 1.9 mg/dL) had an independently increased mortality risk [25]. Although much evidence of CVD and mortality risks due to hypomagnesemia in dialysis patients has accumulated [14, 15], the low-s-Mg level threshold at the start of a hemodialysis session that is likely to cause harm is still unknown. In our study, there was no consistent association between Mg and the incidence of CVD deaths and all-cause mortality, although patients with hypermagnesemia (>2.8 mg/dL) showed the lowest incidence rate of deaths. Supporting our result was a short report that hemodialysis patients with hypermagnesemia >3.0 mg/dL had good nutritional status, low inflammation and a tendency to achieve better survival [26]. Patients with mild hypermagnesemia between 2.7 and 3.0 mg/dL also had the best survival in a Japanese nationwide registry–based cohort study [27]. We speculate that maintaining mild hypermagnesemia at the start of dialysis sessions may contribute to improved mortality, although the upper limit should be managed with caution.

We found a negative linear correlation between s-Mg levels and ERI. A small retrospective study reported an association between anemia and hypomagnesemia in pre-dialysis CKD patients [28]; it also indicated that, in addition to anemia, use of a PPI was another, independent predictor of hypomagnesemia [28]. Over the past decade, PPI prescriptions have increased steadily in elderly hospitalized patients and in CVD patients treated with anticoagulants [29]. It is noteworthy that recent widespread PPI use has contributed to the growing prevalence of hypomagnesemia [30]; dialysis patients are also more prone to Mg deficiency because dietary intake is limited and Mg is removed automatically by dialysis therapy (normally the Mg balance is regulated mainly by the intestines and absorption/excretion takes place without renal regulation) [31]. The additional analysis on s-Mg and ERI suggested that the link between them is not mediated by bone disease and/or inflammation. Although no direct evidence has yet been found to link s-Mg levels to erythropoietin resistance, Mg deficiency is a plausible link to induction of ERI anomalies via risk factor of ERI induced by Mg deficiency discussed above [17, 23, 24]. A report has shown the same correlation between Mg and erythropoietin resistance in hemodialysis patients [18]; this corroborates our results, although the report's s-Mg levels were considerably higher than those in our patients and higher than normal levels [18]. The question whether s-Mg levels could have an impact on clinical outcome in dialysis patients is still unanswered, and the optimal s-Mg level is still unknown.

The strengths of this study are as follows. First, it is the first study to simultaneously investigate the impacts of s-Mg levels on three major dialysis risk factors (anemia, CVD and infectious disease) and mortality. Second, this J-DOPPS-based study was a prospective cohort study with a large sample size that consisted of nationally representative hemodialysis patients undergoing standard maintenance dialysis. Additionally, disorders of s-Mg balance have often been neglected, despite options of any interventional measures with potential impacts, such as diet, supplementation, dialysis prescriptions and cessation of unnecessary PPI. Therefore, evidence linking hypomagnesemia to mortality could lead to new treatment strategies at a low cost and risk [32].

This study has several limitations. First, patients who were not using an ESA at baseline were excluded because their ERI could not be calculated, and a large number of patients were excluded because their Mg value was not available at baseline. The population excluded from analysis due to a missing s-Mg value had a somewhat shorter history of dialysis, but not, in our opinion, sufficient to cause obvious bias in the analyzed population. Second, direct comparison of Japanese s-Mg levels and hemoglobin levels used in ERI calculation with those of other countries was problematic due to differences in sampling timing: in Japanese dialysis centers, blood tests are normally performed during the first dialysis session of the week. Third, we used only one baseline s-Mg in investigating the association with clinical events, which may have hindered precise estimation. Fourth, we did not assess the causal relationships between s-Mg level, ERI and mortality. We also had no data on in whom and why the s-Mg level deviated. Additional international studies are needed to establish a consensus on the ideal s-Mg level in hemodialysis patients.

## CONCLUSIONS

We observed that low s-Mg levels at the start of dialysis session were associated with subsequently worsening erythropoietin resistance in dialysis patients. In addition, we did not detect that low and high s-Mg levels were associated with poor survival. Although further study is needed, establishment of a consensus definition of an optimal s-Mg level has potential to improve outcomes for patients undergoing dialysis.

## Supplementary Material

sfae153_Supplemental_File

## Data Availability

The data underlying this article is not publicly available due to the regulation of J-DOPPS.
