# Supplementary material for: Association between magnesium, erythropoietin resistance and mortality: the Japanese Dialysis Outcomes and Practice Patterns Study (J-DOPPS)
Source: Clin Kidney J. 2024 Jun 21;17(7):sfae153. doi: 10.1093/ckj/sfae153 (PMC11223580; doi:10.1093/ckj/sfae153)
Supplement: sfae153_Supplemental_File [file sfae153_supplemental_file.pdf]

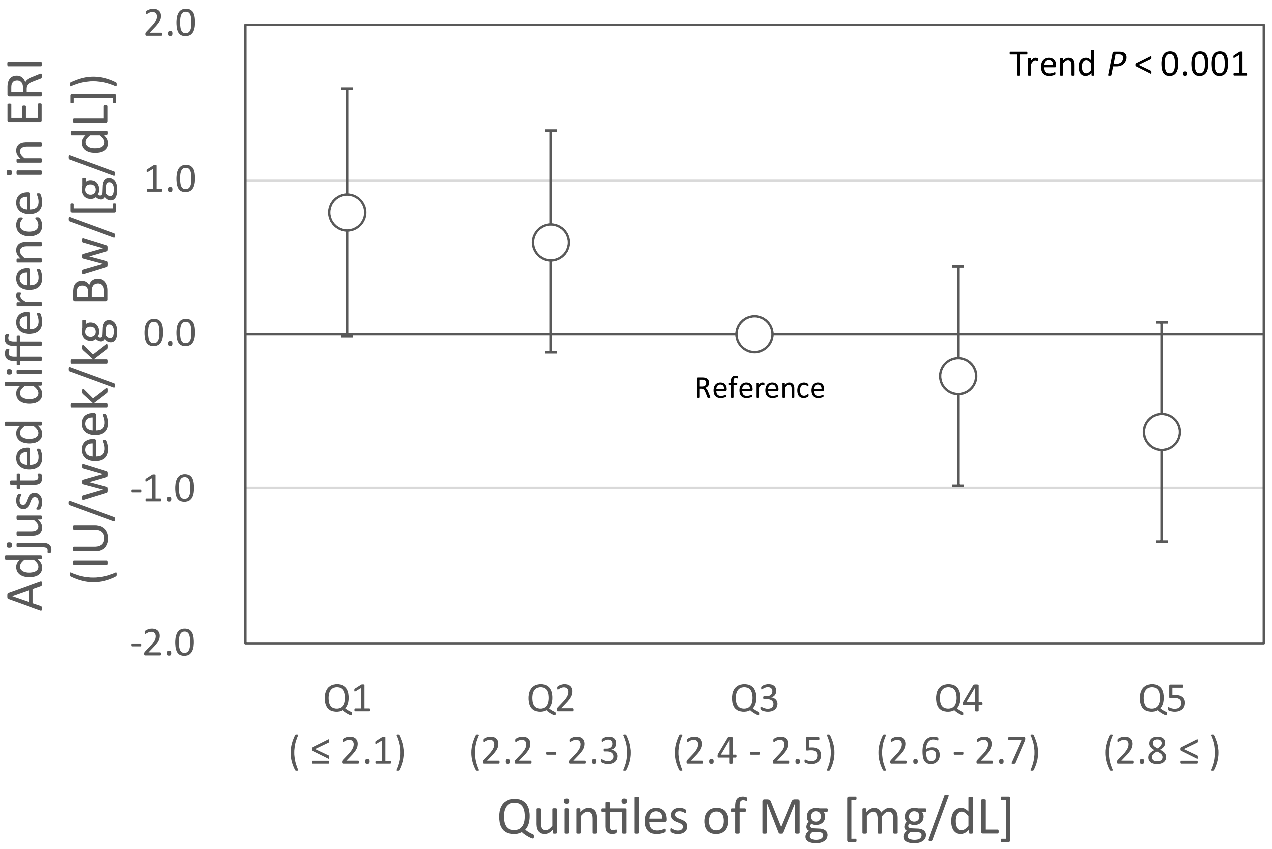

Supplementary Figure 1. A sensitivity analysis on the relationship between erythropoietin resistance index (ERI) and quintiles of serum magnesium (Mg). The regression model included intact parathyroid hormone and C-reactive protein in addition to the main model. Point estimates and 95% confidence intervals of between-group difference with Q3 as the reference are presented.
